# Chronic traumatic encephalopathy (CTE)—features and forensic considerations

**DOI:** 10.1007/s12024-023-00624-3

**Published:** 2023-04-14

**Authors:** Roger Byard, Marianne Tiemensma, Michael E. Buckland, Robert Vink

**Affiliations:** 1 Adelaide School of Biomedicine, The University of Adelaide, Level 2, Room N237, Helen Mayo North, Frome Road, 5005 Adelaide, SA Australia; 2 Forensic Science South Australia, 5000 Adelaide, SA Australia; 3 Forensic Pathology Unit, Royal Darwin Hospital, 0800 Darwin, NT Australia; 4 College of Medicine and Public Health, Flinders University, 5042 Bedford Park, SA Australia; 5 Department of Neuropathology, Royal Prince Alfred Hospital, 2050 Camperdown, NSW Australia; 6 Clinical and Health Sciences, University of South Australia, 5001 Adelaide, SA Australia

**Keywords:** Chronic traumatic encephalopathy, CTE, Neurodegeneration, Tauopathy, Sport, Dementia

## Abstract

Chronic traumatic encephalopathy (CTE) is a neurodegenerative condition, in which the only known cause is exposure to repeated episodes of blunt head trauma. It most often occurs in professional and amateur athletes who have had frequent and repetitive cranial impacts during contact sports, but may also be found in victims of domestic violence, military personnel exposed to explosive devices and in individuals with severe epilepsy. The pathognomonic pathological findings are of neurofibrillary tangles and pretangles in the depths of the cerebral sulci caused by perivascular accumulation of phosphorylated Tau (pTau). Cases may be high profile requiring an evaluation of whether the neuropathological findings of CTE can be related to injuries previously sustained on the sporting field. Failure to examine the brain or to adequately sample appropriate areas at autopsy may lead to cases being overlooked and to an underestimation of the incidence of this condition in the community. Performing immunohistochemical staining for pTau in three areas from the neocortex has been found to be a useful screening tool for CTE. Ascertaining whether there is a history of head trauma, including exposure to contact sports, as a standard part of forensic clinical history protocols will help identify at-risk individuals so that Coronial consideration of the need for brain examination can be appropriately informed. Repetitive head trauma, particularly from contact sport, is being increasingly recognized as a cause of significant preventable neurodegeneration.

## Introduction

Chronic traumatic encephalopathy, or CTE, is a condition characterized by a range of neurological deficits that are attributed to repeated episodes of blunt head trauma. Initially identified in boxers who were considered ‘punch drunk’ with ‘dementia pugilistica’ [1], it has now been found in a much more diverse cohort of individuals including other professional and amateur athletes who have experienced frequent and repetitive concussive and/or sub-concussive impacts during contact sports. It has been noted that former professional/varsity athletes also have a higher rate of other neurodegenerative diseases and neurocognitive disorders [2]. CTE may be found as well in other individuals who have experienced repetitive insults to the brain being documented in isolated reports of victims of domestic violence, military personnel exposed to explosive devices and those with treatment-resistant epilepsy [3-6]. The pathognomonic neuropathological finding is of perivascular accumulation of phosphorylated Tau (pTau) in neurons and neurites, forming neurofibrillary tangles and pretangles initially in the depths of the cerebral sulci [7], but later spreading throughout the brain [8]. The initial perivascular accumulation of pTau at the base of the sulci is thought to be associated with a localized inflammatory response caused by increased physical strains that are focussed on this region from mechanical insults [9].

## Controversies

The condition is not, however, without controversy with assertions being made that there is currently insufficient evidence to support CTE being regarded as ‘a real disease’ [10]. While a consensus statement on concussion in sport in 2016 concluded that there had not been a cause and effect relationship established ‘between CTE and sports-related concussions or exposure to contact sports’ [11], this was in contrast to the opinion from The Centers for Disease Control and Prevention (CDC), in the USA in 2019, that ‘CTE is caused in part by exposure to repeated traumatic brain injuries, including concussion, and repeated hits to the head, called subconcussive head impacts’ [12]. Although some researchers have downplayed the role of repetitive sports-related head injury in causing this specific form of progressive neurodegeneration [13], involvement with sporting organisations and allegations of misinterpretation of data and plagiarism have recently raised concerns of potential researcher bias in reporting and interpretation [14]. The literature, therefore, needs to be evaluated with this possibility in mind; however, although evidence for a connection between repetitive head impacts and CTE has been called ‘imperfect’, there appears no doubt that the two are linked [4].

## Clinical manifestations

Traumatic brain injury is a complex process involving a series of metabolic, neurotoxic, ionic and cellular changes that can lead to progressive tauopathy. CTE is characterized by problems with higher order functions such as multitasking and decision-making with short-term memory dysfunction, emotional instability, apathy and depression [15]. There is also a known association between traumatic brain injury and later-in-life dementia and/or movement disorders such as Parkinson and Alzheimer diseases, with 3–10% of cases of dementia reported to have had previous traumatic cerebral damage [16]. The correlation between clinical diagnoses of dementia and the pathology of CTE identified at autopsy is still unclear, and this has so far prevented accurate assessment of its prevalence. Animal models have, however, been useful in confirming the association between repetitive mild traumatic brain injury and subsequent cognitive impairment with demonstration of increased cortical pTau and microglial activation [17]. The symptoms of CTE are non-specific and progressive, and most victims will not show symptoms until years or decades after the head injury [18]. The diagnosis of CTE is only made postmortem and there is currently no treatment [19].

## Histological features

CTE is a pathological diagnosis, defined by aggregates of phosphorylated Tau in neurons, with or without thorn-shaped astrocytes. These are located in perivascular areas in the depths of sulci, not restricted to the subpial and superficial areas of the cortex, but also deeper in the parenchyma [6, 20]. Criteria have been developed for the histologic diagnosis of CTE listing both required and supportive features, with specification of minimal thresholds for making the diagnosis. Further details may be found in the 2021 consensus statement by Bieniek et al. [21].

## Forensic issues

Determining whether the neuropathological features of CTE are present at autopsy, or not, may now have considerable medicolegal significance if a link is attempted to be established between injuries sustained on the sporting field or elsewhere and subsequent neurodegenerative changes with behavioural sequelae. Issues that may arise include those of duty of care at sporting events and the quality of post-concussive clinical management and time of return to play. As there has been a purported link between CTE and risk of suicide, this may represent another avenue that may be explored in a legal context. The association of CTE with aggressive behaviour has also been raised in a case where an affected individual has been involved in a homicide [1, 22]. While it is beyond the purview of a forensic pathologist to comment on the possible or likely behavioural manifestations of CTE, it may be a requirement to have performed or arranged an appropriate neuropathological evaluation to establish whether the typical pathological features were present or not. In a forensic context, CTE is most often coincidental to the cause of death with an individual dying with, rather than from, it, as deaths directly relating to sporting activities are more often acute due to accidents or congenital or acquired cardiovascular disease [23].

Problems that arise with the diagnosis of CTE include the lack of routine sampling of brain tissue in target areas at autopsy and the absence in most forensic laboratories of facilities for immunohistochemical staining. In addition, few forensic pathologists have formal neuropathology training, as it is a very highly specialized area. Under the best conditions, a neuropathology service will be available which can either process the brain completely or provide guidance on which areas to sample. Also, a specialist laboratory will have the range of stains that are needed, in addition to a neuropathologist who can interpret the subsequent findings.

**Fig. 1:**
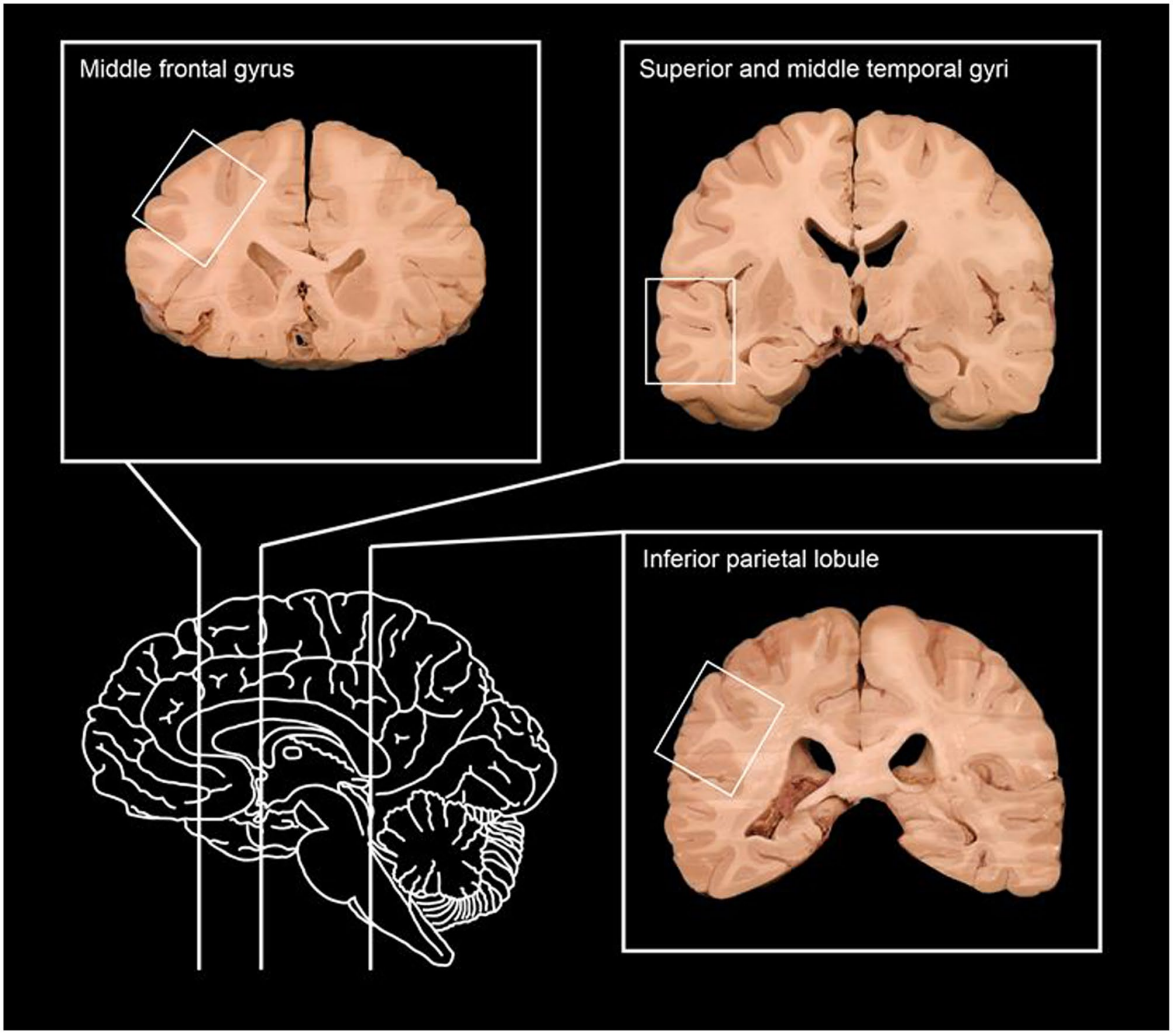
The three cortical areas with the highest yield for CTE screening (from - https://www.neuroanatomy.ca/index.html -This work is licensed under a *Creative Commons Attribution-NonCommercial-ShareAlike 4.0 International License*)

As it has been demonstrated that performing immunohistochemical staining for pTau in three areas from the neocortex is a useful screening tool for CTE, perhaps this should be made part of routine sampling at autopsy to enable retrospective assessment if it is later sought? The three highest yield areas for CTE screening described in the 2021 consensus statement [21] are as follows:

- The middle frontal gyrus
- The inferior parietal lobule
- The superior and middle temporal gyri (Fig. 1)

A slightly modified version of this screening protocol has been used in Australia, with the identification of four cases of CTE that may have been otherwise overlooked over a 3-year-period [6]. In cases of suspected CTE, a more extensive sampling protocol should be undertaken [21], or the brain referred to specialist centre for examination.

Of course, much of this is predicated on the diagnosis of CTE being suspected prior to autopsy and on the inclusion of brain examination as part of the postmortem. Increasingly in the era of postmortem imaging, the cranial cavity is not being routinely opened if no abnormalities are seen on CT examination, with the examinations being limited to either external or targeted thoracic or abdominal cavity inspections/dissections. This may result in further cases not being identified and in reduced numbers being documented in the postmortem forensic population. A recent Australian Coronial inquest into a high-profile suicide has recommended that Coronial processes be modified to “Improve timely identification of cases in which there is a history of head trauma, be that major trauma or minor repetitive trauma, such as may be sustained in sporting activities, so that consideration of the need for an autopsy can be appropriately informed” [24]. Therefore, a history of contact sports participation should be included when police/coronial counsellors are gathering information, particularly if there have been indicators of behavioural/mood issues and/or suicide.

## Conclusion

CTE is a definite neuropathological entity with quite characteristic histological features and relatively protean clinical manifestations. The link with repetitive head trauma, including that associated with sporting activities has been established. However, determining the clinical course and incidence of the disorder, in addition to establishing stronger clinicopathological predictors and clarifying epidemiological characteristics, may be problematic [25, 26]. This process will be influenced by the particular medicolegal focus of cases that present for forensic examination in different jurisdictions, the availability of specialist neuropathological services, the numbers of autopsy cases that have cranial cavity and brain examinations, the numbers of hospital autopsies being undertaken and finally on the numbers of cases with relevant and complete clinical information [16, 27].

As it has been estimated that there are at least 1.6 to 3.8 million sport/recreational related-concussions per year in the USA, with 73% of retired professional footballers in Australia having at least one episode of concussion over their careers (over half with multiple episodes) [28], it is possible that there are a number of occult cases currently in the community. Given this possibility, CTE is a condition that will in all likelihood command increasing forensic attention in future years.
